# Homogeneous Space Construction and Projection for Single-Cell Expression Prediction Based on Deep Learning

**DOI:** 10.3390/bioengineering10090996

**Published:** 2023-08-23

**Authors:** Chia-Hung Yeh, Ze-Guang Chen, Cheng-Yue Liou, Mei-Juan Chen

**Affiliations:** 1 Department of Electrical Engineering, National Taiwan Normal University, Taipei 10610, Taiwan; j253069@gmail.com (Z.-G.C.); a795138865yue@gmail.com (C.-Y.L.); 2 Department of Electrical Engineering, National Sun Yat-sen University, Kaohsiung 80424, Taiwan; 3 Department of Electrical Engineering, National Dong Hwa University, Hualien 97401, Taiwan

**Keywords:** cell perturbation response prediction, disentangled representations, cell response homogeneous space construction, deep learning, interpretability

## Abstract

Predicting cellular responses to perturbations is an unsolved problem in biology. Traditional approaches assume that different cell types respond similarly to perturbations. However, this assumption does not take into account the context of genome interactions in different cell types, which leads to compromised prediction quality. More recently, deep learning models used to discover gene–gene relationships can yield more accurate predictions of cellular responses. The huge difference in biological information between different cell types makes it difficult for deep learning models to encode data into a continuous low-dimensional feature space, which means that the features captured by the latent space may not be continuous. Therefore, the mapping relationship between the two conditional spaces learned by the model can only be applied where the real reference data resides, leading to the wrong mapping of the predicted target cells because they are not in the same domain as the reference data. In this paper, we propose an information-navigated variational autoencoder (INVAE), a deep neural network for cell perturbation response prediction. INVAE filters out information that is not conducive to predictive performance. For the remaining information, INVAE constructs a homogeneous space of control conditions, and finds the mapping relationship between the control condition space and the perturbation condition space. By embedding the target unit into the control space and then mapping it to the perturbation space, we can predict the perturbed state of the target unit. Comparing our proposed method with other three state-of-the-art methods on three real datasets, experimental results show that INVAE outperforms existing methods in cell state prediction after perturbation. Furthermore, we demonstrate that filtering out useless information not only improves prediction accuracy but also reveals similarities in how genes in different cell types are regulated following perturbation.

## 1. Introduction

Due to the development of single-cell RNA-seq (scRNA-seq) technology, we are able to record gene expression levels in single cells [1]. Single-cell RNA-seq is used by researchers in various fields, such as development [2,3], immunity [4], and drug response [5,6]. However, the cost of obtaining scRNA-seq is still high, because the combinations of different cell types and drugs or infectivity are so large that it is almost impossible to measure the cellular response in each situation. Furthermore, some data, such as testing patients for unlicensed drugs, are difficult to obtain [7]. In contrast, using computational methods to predict how cells will respond to different conditions is less expensive. Furthermore, predictive unobserved analytics can facilitate drug repurposing or individualized treatment [7].

Deep learning has shown promising performance in many fields including bioinformatics [8,9], and can discover highly nonlinear relationships in high-dimensional data. In recent years, many deep model-based studies on cellular response prediction have been proposed. The key to prediction is the correct projection of target cells from one condition to another. scPreGAN [10] and TSPG [11] use generative adversarial networks (GAN) [12] to generate predicted cellular responses. They use the discriminator to encourage the generator to project the data into the desired space, and the data fed into the discriminator is the generated data. stVAE [13], trVAE [14], and CPA [15] use the concept of the conditional variational autoencoder (CVAE) [16] to generate specific data because it allows latent variables to be distributed under specific conditions, rather than the entire dataset. By inserting condition-related variables into scRNA-seq and latent variables, the encoder can filter out changes caused by different conditions. The model learns how different conditions vary, so it is feasible to project the data to different conditions. To further force the model to find a more correct feature projection from one condition to another, stVAE and CPA use a discriminator to encourage the model to filter out biological state information.

Instead of using a discriminator, trVAE adds a maximum mean difference (MMD) [17] loss to force the encoder to encode data as condition-independent latent variables, avoiding the well-known min/max problem of adversarial models. MichiGAN [18] combines variational autoencoder (VAE) based models [19] and GAN-based models. Using β-TCVAE [20] to learn a disentangled representation and feed the disentangled latent variables into a GAN maintains high generation quality, while the generated results can be controlled by changing the value of the disentangled latent variables. Assuming cell-type latencies are homogeneous across conditions, unseen perturbations in cell-type gene expression can be predicted by adding differential latency values across cell types and conditions. This paper proposes an information-navigated variational autoencoder, called INVAE, which can predict unseen cell states after perturbations. To force the model to learn an uncorrelated and non-informatively redundant latent space, we adopt β-TCVAE instead of plain VAE. INVAE navigates condition-invariant and condition-specific information to different parts of latent variables. INVAE can make predictions more accurate by filtering out information that is irrelevant to predictions. In addition, INVAE can also discover whether different cell types or species have similar drugs or infection mechanisms.

The rest of the paper is organized as follows. Section 2 describes the details of the proposed framework. The testing datasets and the setting of benchmark methods are introduced in Section 3. Experimental results are demonstrated in Section 4. Finally, concluding remarks are made in Section 5.

## 2. Materials and Methods

### 2.1. Problem and Theory Explanation

The problem of cell perturbation response prediction is described as follows: Suppose we have a set of scRNA-seq data (target data) containing one cell type and only one condition (control), and the goal is to predict the unseen perturbation condition in another cell with the same gene set and a dataset of different cell types (reference dataset). In order to accurately predict cellular responses, the model must accomplish two tasks: 1. The model must decompose the data information with the same condition into multiple manifolds. 2. The model must learn the precise projective relationship between these manifolds from one condition to another. After the model is successfully built, the model can be used to project the data into the perturbation condition space and output the prediction data, so that the perturbation response of unseen cells can be predicted. Although the reference dataset contains information for the model to accomplish tasks 1 and 2, not all information provided in the reference dataset is necessary to build the desired model. Some features, such as the conditional invariance of gene sets, remain constant across conditions, but these should be considered as noise that may reduce prediction accuracy. This paper presents an INVAE model that aims to predict cellular perturbation responses by accomplishing the above two tasks. Since MichiGAN has demonstrated that β-TCVAE can learn disentangled meaningful biological information, we adopt β-TCVAE instead of ordinary VAE to extract features from data.

### 2.2. VAE & β-TCVAE

Like autoencoders (AE) [21], VAEs have encoders and decoders; however, instead of encoding data into deterministic latent codes, VAEs encode data into a multivariate normal distribution. Specifically, the VAE’s encoder produces mean and standard deviation outputs to represent the data distribution in the latent space. The distribution of the latent space is a Gaussian mixture model consisting of the distribution encoded by the data. The overall distribution of the latent space is regularized to approximate a standard multivariate normal distribution. The VAE’s decoder then samples the latent codes from the latent space to reconstruct the original data. The loss of VAE is shown in the equation below:

(1) $$L_{vae}=E_{q\varphi (z|x)}\;[log\;p_{\theta }(x|z)]-\beta D_{KL}(q_{\varphi }(z|x)||p\left(z\right)),$$

where $q_{\varphi }\;$ is the encoder, $p_{\theta }$ is the decoder, x is the input data, and z is the latent space; $p\left(z\right)\;$ denotes the standard multivariate normal distribution. The first term is the reconstruction loss, and the second is the Kullback–Leibler (KL) divergence, which encourages the distribution of the latent space to approximate a standard multivariate normal distribution, with the coefficient β set to 1 for the KL divergence. β-TCVAE decomposes the KL divergence loss in VAE into three terms, and the equation is as follows:

(2) $$\begin{array}{l} D_{KL}(q_{\varphi }(z|x)||p\left(z\right))\\ \;\;\;\;\;\;=D_{KL}[q_{\varphi }(Z,X)||q_{\phi }(Z)q(X)]+D_{KL}[q_{\varphi }(Z)||\prod_{k}q_{\varphi }(Z_{k})]\\ \;\;\;\;\;\;+\sum_{k}D_{KL}[q_{\varphi }(Z_{k})||p(Z_{k})]. \end{array}$$

The first term, total correlation (TC), measures the correlation between latent variables and is the most effective of the three to disentangle the data-generating factors. Our results show that β-TCVAE allows higher tuning of the hyperparameters for total correlation than previous methods and achieves better disentanglement rates without degrading reconstruction quality too much.

### 2.3. Assumptions

In any given scRNA-seq dataset, annotations such as cell type and condition are denoted as c and s. We assume the existence of a mapping function that partitions cell information into two spaces: the condition-invariant space ${z_{c}\in Z}_{c}$, where features remain unchanged after perturbations, and the condition-specific homogeneous space ${z_{s,h}\in Z}_{s,h}$, which learns homogeneous information across different cell types. Here represents the projection of a homogeneous space between different conditions s, where h represents different latent variables. After finding projections of homogeneous spatial relationships between different conditions, it is possible to predict how cells will respond to different perturbations.

### 2.4. Overview of INVAE

**Figure 1:**
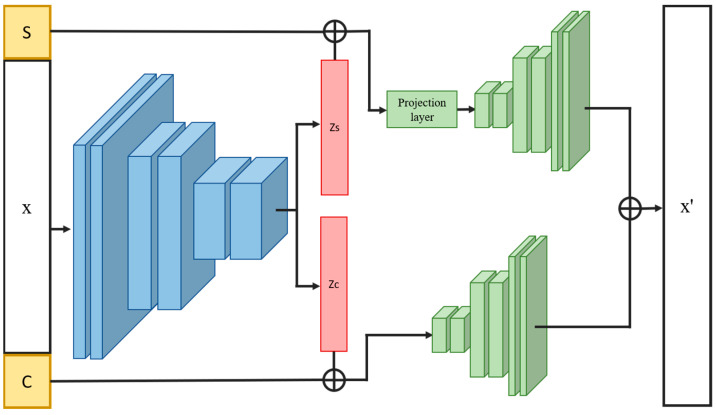
Overview of the proposed architecture. The encoded latent variables are split into two parts and fed into corresponding decoders. The input data are then reconstructed by summing the two outputs of the two decoders. Symbols c and s are the corresponding variables determined by cell type and data condition, respectively.

The proposed INVAE is a cellular perturbation response prediction model that aims to identify precise projective relationships between different conditions in feature space by filtering out irrelevant correlations. In our approach, the features of latent variables are first divided into two parts: condition-invariant features and condition-specific features. As shown in Figure 1, the architecture of the INVAE model consists of three components: an encoder, a projection layer, and two decoders. Encoders are used to extract features from data. The relative loss function is the same as β-TCVAE.

To analyze the condition-invariant characteristics of the dataset, we divided the whole dataset into several sets according to different cell types by the ANOVA (analysis of variance) algorithm. ANOVA is an algorithm that can determine whether data sets consist of the same distribution based on the latent variables of each dataset. By reducing the values determined by the results of tests run on each group, we can identify those condition-invariant features that do not change after perturbation. To predict unseen cell responses to perturbations, condition-specific features of variables must separate condition-invariant from condition-specific features. Likewise, we selected the data belonging to the control condition and treated them as one group, and divided this group of data into different groups according to different cell types. Finally, we performed ANOVA for testing. To reduce the test value of ANOVA, encoders were used to look for homogeneous information between different cell types.

**Figure 2:**
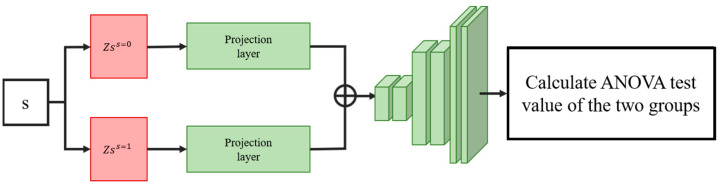
Illustration of the loss calculation of the projection layer. Condition-specific features of latent variables with the same cell type should be projected to the same distribution by the projection layer if the interpolating variables are the same. By doing so, the model is forced to find the correct projective relationship for different conditional spaces.

Condition-specific features of the latent variables are then concatenated by inserting hard-coded variables according to the condition of the data. This variable is used to control the conditional state of the generated data. As shown in Figure 2, we assign the variables encoded by the same cell type data into the same set and divide each set into two groups according to different conditions to find the projection relationship of different conditional homogeneous spaces. To condition the output of a condition-specific feature by inserting variables, we insert the same variable (control variable or disturbance variable for two different ANOVA tests) into both groups and input this set of variables into the projection layer, and each variable output by the projection layer is tested by analysis of variance. Restricting the projection layer will ensure that the model learns the projection relationship of different conditional spaces.

To lower the test value of the ANOVA, we first execute the model to test the output variables of the encoder and projection layers. The results generated by conditional space learning of uncorrelated filtering, homogeneous space construction, and projective relations are then used for cellular response prediction.

The conditional invariance feature of variables is combined with interpolation variables based on the cell type of each data. This process allows the model to encode information about variation across cell types as conditional invariance of variables. Here, the concatenated variables will be fed to decoder 1. The condition-specific features of the variables and the interpolation variables mentioned above will be fed to decoder 2. The sum of the outputs of decoder 1 and decoder 2 will be used to recover the original data.

### 2.5. Loss Functions

#### 2.5.1. β-TCVAE Loss

As mentioned above, β-TCVAE is used to extract features from data while ensuring that the learned information is separated from each other. We separate reconstruction loss for target data from reconstruction loss for other data to train more accurate predictive models. Overall, the β-TCVAE loss is defined as follows:

(3) $$L_{TCVAE}=L_{recon\_other}+\beta L_{TC}+L_{KL},$$

where β is set to 2 to increase the disentanglement of latent variables and $L_{recon\_other}$ is the reconstruction loss for input data other than target data; $L_{TC}$ and $L_{KL}$ are the TC loss and KL loss, respectively.

#### 2.5.2. ANOVA Information Navigation Loss

To control the distribution of latent variables to further navigate specific information into specific latent variables, we use ANOVA as the loss function. ANOVA loss functions are designed to enable uncorrelated filtering, homogeneous space construction, and learning of conditional space mapping. The ANOVA loss is defined as follows:

(4) $$L_{ano}(X,I,J)=\sum_{i}^{I}ANO(X_{i,J}),$$

where I and J are sets of data annotations such as cell type or condition, and $X_{i,J}$ is the set of data annotated as i and divided into several groups by annotations in set J. $ANO(X_{i,J})$ is an ANOVA test on $X_{i,J}$. The smaller the value, the higher the probability that each group of data comes from the same distribution.

#### 2.5.3. Total Loss Function

The total loss of INVAE is of two types, depending on the settings of the training procedure elaborated in Section 2.6 and defined as follows:

(5) $$L_{total}=L_{TCVAE}+\;L_{ano}\left(Z_{c},C,S\right)+L_{ano}\left(Z_{s},C,S_{con}\right)+L_{ano}\left(Z_{p},C,S\right),$$

or:

(6) $$L_{total}=L_{TCVAE}+L_{ano}\left(Z_{c},C,S\right)+L_{ano}\left(Z_{s},C,S_{con}\right)+L_{ano}\left(Z_{p},C,S\right)+L_{recon\_target},$$

where $Z_{c}$ is the first part of latent variables, $Z_{s}$ is the second part of latent variables, S and C are the condition set and cell type annotation, respectively. $L_{recon\_target}$ is the reconstruction loss of the target cell, $S_{con}$ is a set with only one element (control condition), $Z_{p}\;$ is the output variable of the projection layer, and the set of input variables is composed of the methods we mentioned above.

### 2.6. Training Processes

The key to cell response prediction in INVAE is the construction of a homogeneous space and the corresponding spatial mapping under different conditions. For different conditional spatial mappings, the model can be trained using cell-type data with control conditions and perturbation conditions to learn the correct spatial mapping relationship. However, the target data has only one condition type; thus, the correctness of different conditional mappings may suffer when the model tries to impose information from the target data into the latent space. Here, inspired by transfer learning [22], a training procedure is designed to reduce the damage to the target data caused by learning different conditional mappings. Transfer learning is a method in which a learning task is used to train a model and then the trained model is applied to solve a testing task. One of the popular applications of transfer learning is to find learning tasks that have similar characteristics to the test task, so a model trained on the learning task can solve the test task by fine-tuning the model with the test task because the trained model on the learning task can also capture characteristics of most testing tasks.

Here, the reference unit and the whole dataset can be regarded as the learning task and the testing task, and we use the reference dataset to train the model, which can also capture most of the characteristics of the target data. The difference is that the learning task contains the entire dataset including the target data, but the loss function does not contain $L_{recon\_target}$. Training on a learning task allows the model to use the target data to build a homogeneous space in which it can be embedded, rather than attempting to impose specific information about the target data in the latent space. After the model has completed the training process for the learning task, instead of fine-tuning on the test task (Equation (5) as the loss function), we use Equation 6 to update the model every N iterations and set N to 5 in this paper. The overall training process is shown in the Algorithm 1 below.
**Algorithm 1:** INVAE training process Input: data, X; annotation of cell-type and condition, C, S; encoder, F(.); decoder1 and 2, $G_{1}(.),\;G_{2}(.)$; projection layer, P(.) 1. count = 0 2. While FinishTraining! = True: 3.   For b in numBatches: 4.     Get training batch x, c, s, from X, C, S 5.     Z = F(concat(x, c, s)) 6.     Split latent variables Z into first and second parts: $Z_{c}$, $Z_{s}$ 7.     ${x^{\prime }}_{c}$ = $G_{1}(concat(Z_{c},c)$) 8.     ${x^{\prime }}_{s}$ = $G_{2}(P(concat\left(Z_{s},s\right))$ 9.     For i in {i: i ∈ c}: 10.       s ∈ {0, 1} 11.       Get $Z_{1}$ from $Z_{s}$ which annotation is (c = i) & (s = 0) 12.       Get $Z_{2}$ from $Z_{s}$ which annotation is (c = i) & (s = 1) 13.       Create set $D_{1}$ = {$P(concat\left(Z_{1},0\right))$*,*
$P(concat\left(Z_{2},0\right))$} 14.       Create set $D_{2}$ = {$P(concat\left(Z_{1},1\right))$*,*
$P(concat\left(Z_{2},1\right))$} 15.     End 16.     Calculate (5), (6) with Z, $Z_{c}$, $Z_{s}$, ${x^{\prime }}_{c}$, ${x^{\prime }}_{s}$, x, $D_{1}$, $D_{2}$ 17.     If count < N: 18.       Update model using (5) 19.     elif count == N: 20.       Update model using (6) 21.       count = 0 22.     End 23.     count += 1 24.   End 25. End

## 3. Datasets and Benchmarks

For evaluation, we compared our model with three baseline models tested on three real datasets. For each dataset, we trained a model by removing perturbed condition data for one cell type and used the model to predict the gene distribution of perturbed cells based on the control condition for that cell type. The details of the benchmarks and datasets are described below. In addition, the following datasets and algorithm codes were accessed on 1 September 2021.

### 3.1. Haber Dataset

The Haber dataset [23] contains eight types of intestinal cells with three conditions: healthy, infected with *Heligmosomoides polygyrus* (*H. poly*), and infected with Salmonella. We downloaded the preprocessed dataset from (github.com/theislab/trvae_reproducibility), and retained and normalized 2000 highly expressed genes.

### 3.2. Kang Dataset

The Kang dataset [24] consists of eight types of control cells and interferon-β (IFN- β)-stimulated human peripheral blood mononuclear cells. The dataset was downloaded from (github.com/theislab/trvae_reproducibility), and 2000 highly expressed genes were retained and normalized.

### 3.3. LPS Dataset

The LPS dataset [25] contains phagocytosis data for 6619 genes from four species (mouse, pig, rabbit, and rat). The dataset includes two conditions: control and lipopolysaccharide (LPS) interference for h. The dataset we used was downloaded from (https://github.com/theislab/scgen-reproducibility/blob/master/code/DataDownloader.py). We selected 2000 highly variable genes consistent with the above.

### 3.4. trVAE

For the Haber and the Kang datasets, we used the reproducible code (github.com/theislab/trvae_reproducibility) proposed by the authors without changing any hyperparameters the author set to train the two datasets. For the LPS dataset, we used the same downloaded code with the default hyperparameters set in their model. In addition, trVAE [14] calculates the Pearson correlation to measure the performance of the prediction of cell perturbation responses.

### 3.5. stVAE

We downloaded stVAE from (https://pypi.org/project/stVAE/). Except for the input dimension, other hyperparameters remain default.

### 3.6. scPreGAN

We downloaded the source code from (https://github.com/JaneJiayiDong/scPreGAN-reproducibility). Architecture and hyperparameters remain default.

### 3.7. Hyperparameter

We keep the hyperparameters of our proposed model consistent across all datasets to ensure training consistency. Table 1, Table 2, Table 3 and Table 4 illustrate these hyperparameters, illustrating the specific configuration of the model to give a comprehensive understanding of our experimental setup.

## 4. Results

### 4.1. Prediction Accuracy Comparison

**Figure 3:**
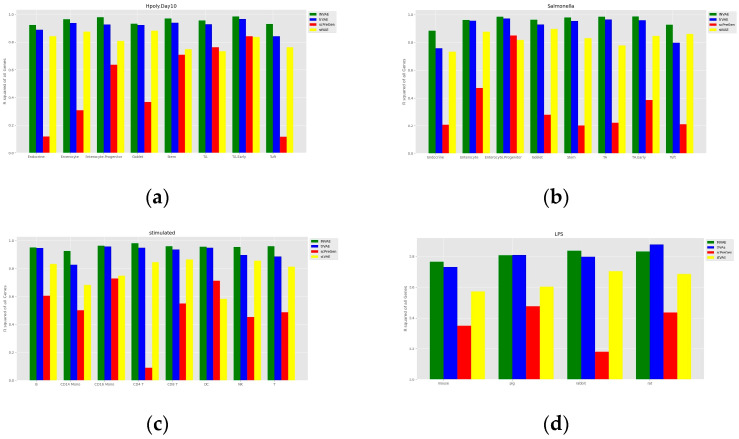
Comparisons of the R-squared value between the average expression level of genes in real perturbed cells and the average expression level of genes in predicted cells. (**a**) Comparison of H.poly results for the Haber dataset. (**b**) Comparison of Salmonella results for the Haber dataset. (**c**) Comparison of results for the Kang dataset. (**d**) Comparison of results on the LPS dataset.

**Figure 4:**
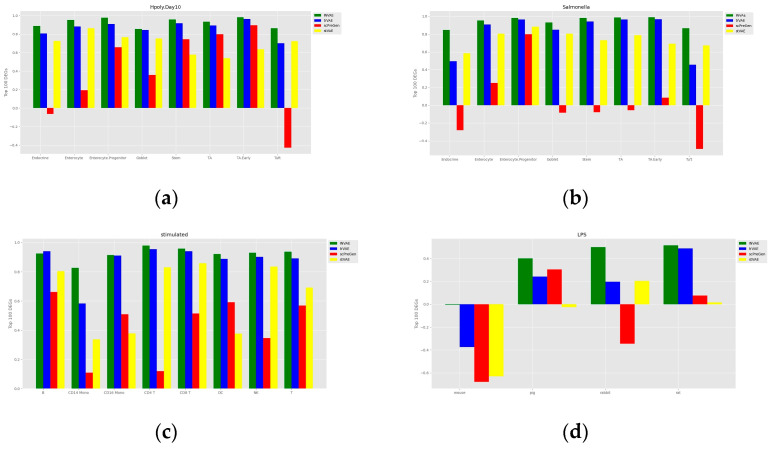
Comparisons of the R-squared value between the average expression level of the top 100 genes in the DEGs of the real perturbed cells and the average expression level of the top 100 genes in the DEGs of the predicted cells. (**a**) Comparison of H.poly results for the Haber dataset. (**b**) Comparison of Salmonella results for the Haber dataset. (**c**) Comparison of Kang dataset results. (**d**) Comparison of results on the LPS dataset.

To assess the accuracy of the prediction of cell perturbation responses, we calculated the R-squared value between the average expression level of genes in real perturbed cells and the average expression level of genes in predicted cells. To further assess the model’s ability to predict highly differentially expressed genes (DEGs), we also calculated the R-squared value between the real and predicted data for the top 100 DEGs for each cell type. A comparison of the models is shown in Figure 3 and Figure 4. The proposed INVAE can accurately predict cellular perturbation responses for most cell types and perturbations. For these three benchmarks, the R-squared values for the top 100 DEGs dropped dramatically compared to the values for all genes used in some cell-type-specific predictions (e.g., Tuft cells, CD14 Mono, and LPS datasets); however, INVAE does not drop that much. This suggests that INVAE is better able to capture the responses of highly variable genes because INVAE creates a homogenous feature space that can efficiently discover similarities. All four models performed significantly worse on the LPS dataset compared to the other datasets, possibly because the cross-species information was too large for the models to have enough biological information to make good predictions. Nonetheless, INVAE captured the most DEG responses compared to the other three models.

### 4.2. Interpretability

**Figure 5:**
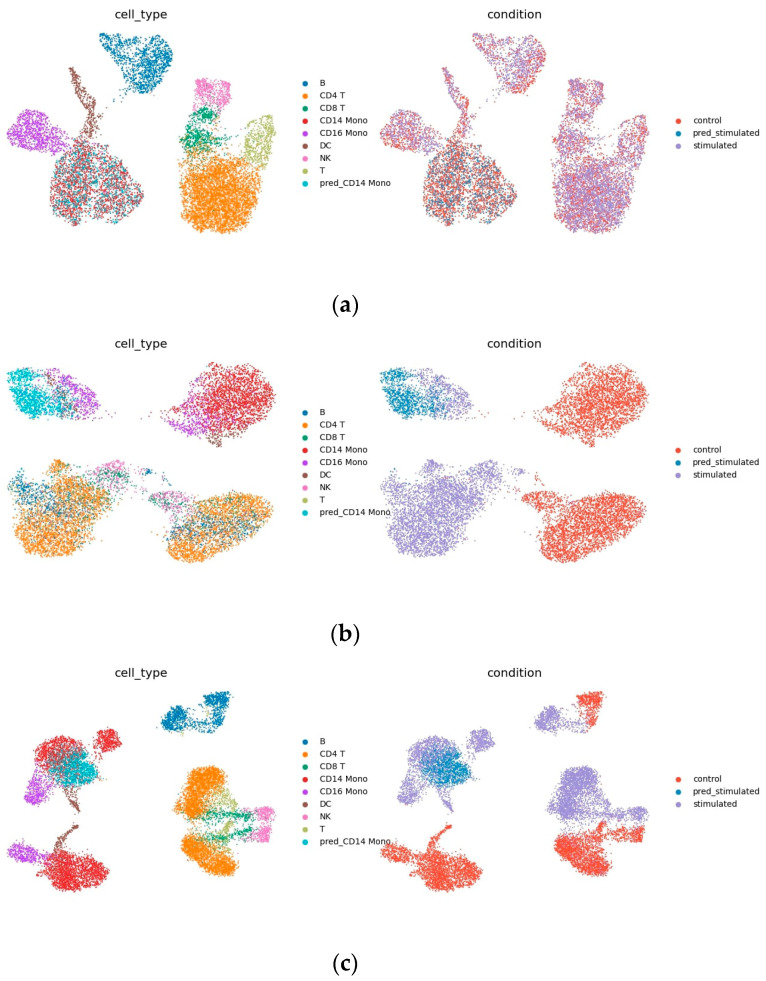

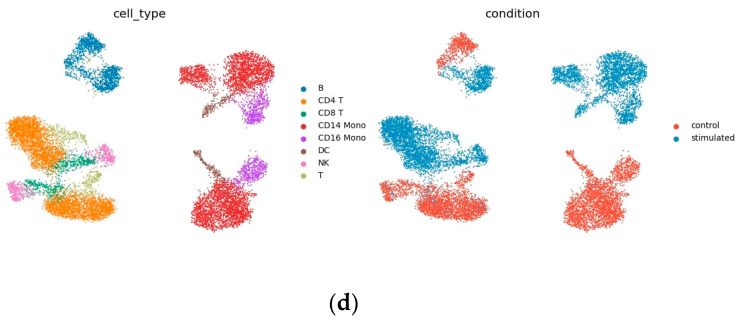
Uniform manifold approximation and projection (UMAP) visualization: (**a**) Decoder 1 trained to predict the output of CD14 Mono perturbation, which is the decoding result of condition invariant space; (**b**) decoder 2 trained to predict CD14 mono perturbation, which is the decoding result of condition invariant homogeneous space, (**c**) CD14 mono (sum of outputs of decoder 1 and 2) and prediction results of Kang dataset, (**d**) Kang dataset.

Figure 5a,b show the output of decoders 1 and 2. As shown in Figure 5a, in the decoded output of the condition-invariant space, data from different conditions have the same cell type overlap, indicating that this part only contains differences resulted from different cell types. Figure 5b shows the decoded output of the homogeneous space, as shown, after filtering out the condition-invariant part, the remainder shows similarities in the perturbation responses of different cell types not observed in the raw data (Figure 5d). For example, it is immediately clear that B cells and CD4 T cells respond similarly to IFN-β stimulation (Figure 5b), but cannot be read from the raw data.

Note that the ability to see similarities in cell responses also makes predictions easier to interpret, and the idea of INVAE is to embed target cells in a homogeneous space and project them onto other conditional spaces. By visualizing the decoded output of the homogeneous space, we can check whether the target unit has been successfully or reasonably embedded in the homogeneous space. If the output from visualizing a homogeneous space looks strange—for example, if cell types with large differences in biological information should be mixed together, or if predicted cells are embedded in strange places—we might suspect that the quality of the prediction is poor. Compared to other methods that directly generate predictions, the homogenous space generated by INVAE filters condition-invariant information, which allows us to check whether the predictions are convincing, avoiding the notorious black-box problem of deep models.

### 4.3. INVAE Captures Non-Linearly Gene–Gene Interaction Features

One of the reasons why deep learning methods have shown amazing performance in various fields is their ability to automatically capture nonlinear relationships in high-dimensional data. The four cellular response prediction methods presented here are based on deep learning models designed to exploit their ability to capture features of nonlinear gene–gene interactions to make accurate predictions. Among the four methods, INVAE can best capture gene–gene interaction features, resulting in the highest prediction accuracy, as shown above (see Figure 3 and Figure 4). We visualize the distribution of some selected genes to further demonstrate this question.

**Figure 6:**
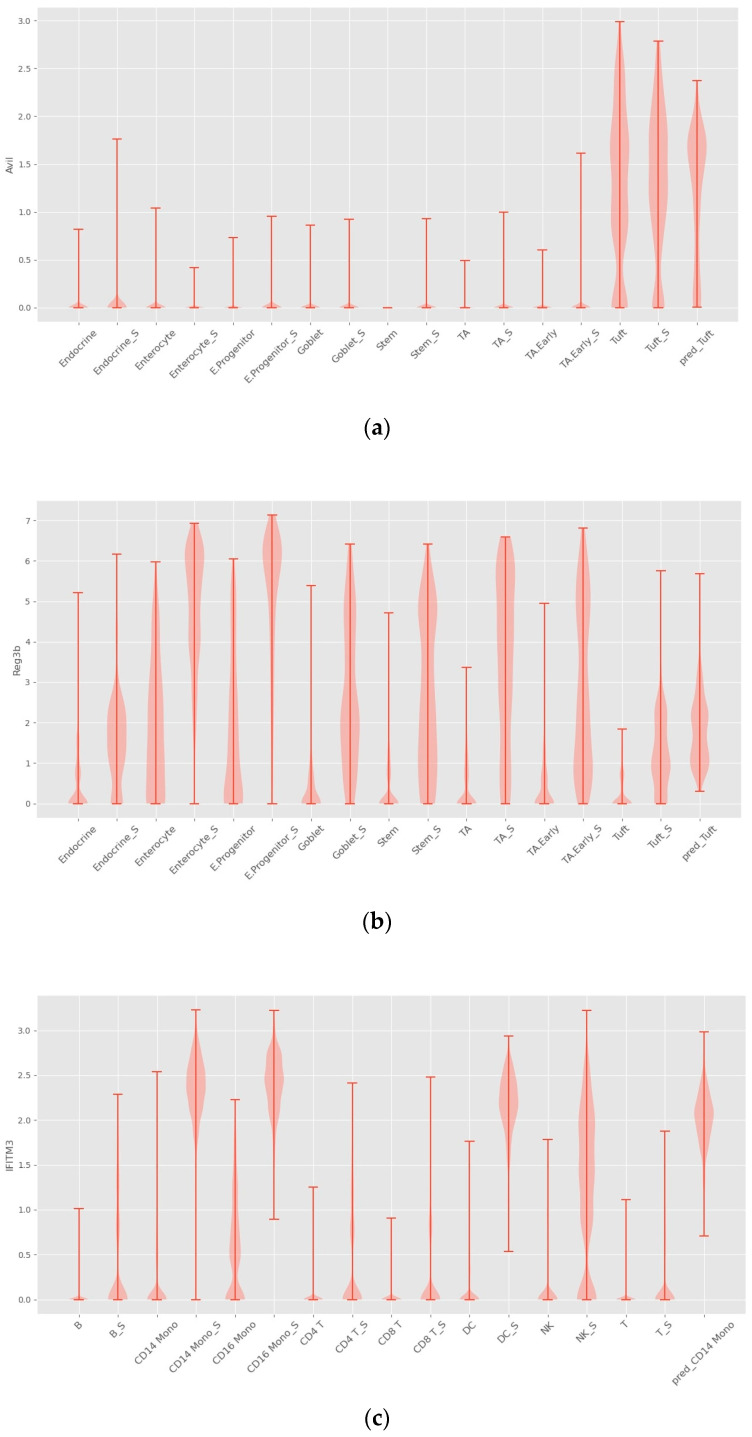

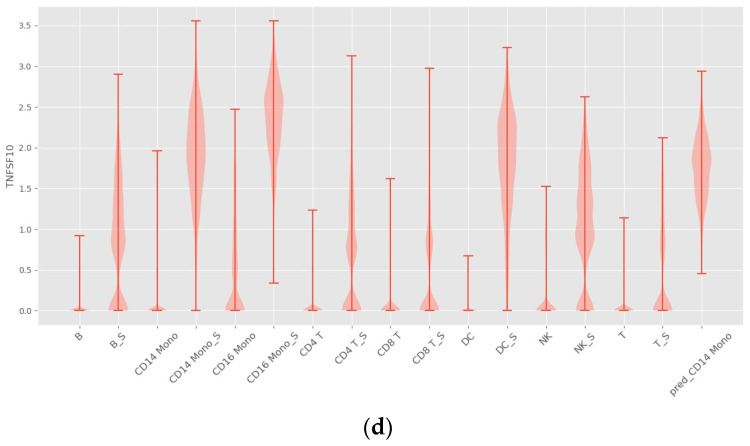
Violin plots with random sampling of true cell type gene distributions and INVAE predicted gene distributions in the dataset, cell names with the suffix “S” indicate perturbation conditions. (**a**) Violin plot of *Avil* in the Haber dataset. (**b**) Violin plot of *Reg3b* in the Haber dataset. (**c**) Violin plot of *IFITM3* from the Kang dataset. (**d**) Violin plot of *TNFSF10* in the Kang dataset.

As shown in Figure 6, INVAE can predict whether the target cell has the same gene expression level as the reference cell, because the prediction made by the model based on the characteristics is composed of multiple genes, not just a gene expression level.

**Figure 7:**
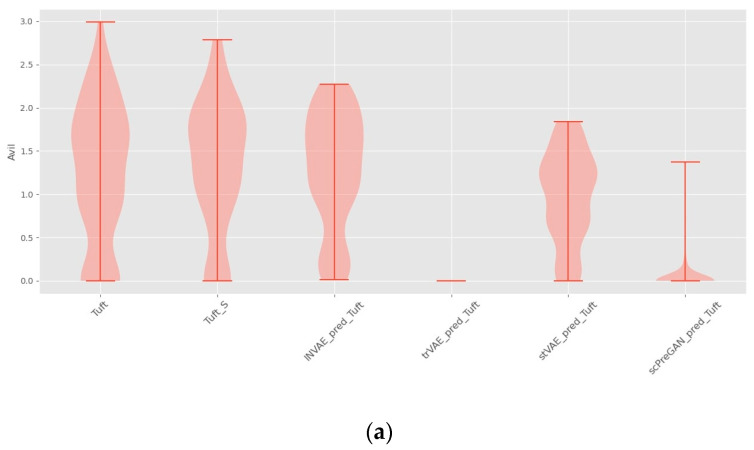

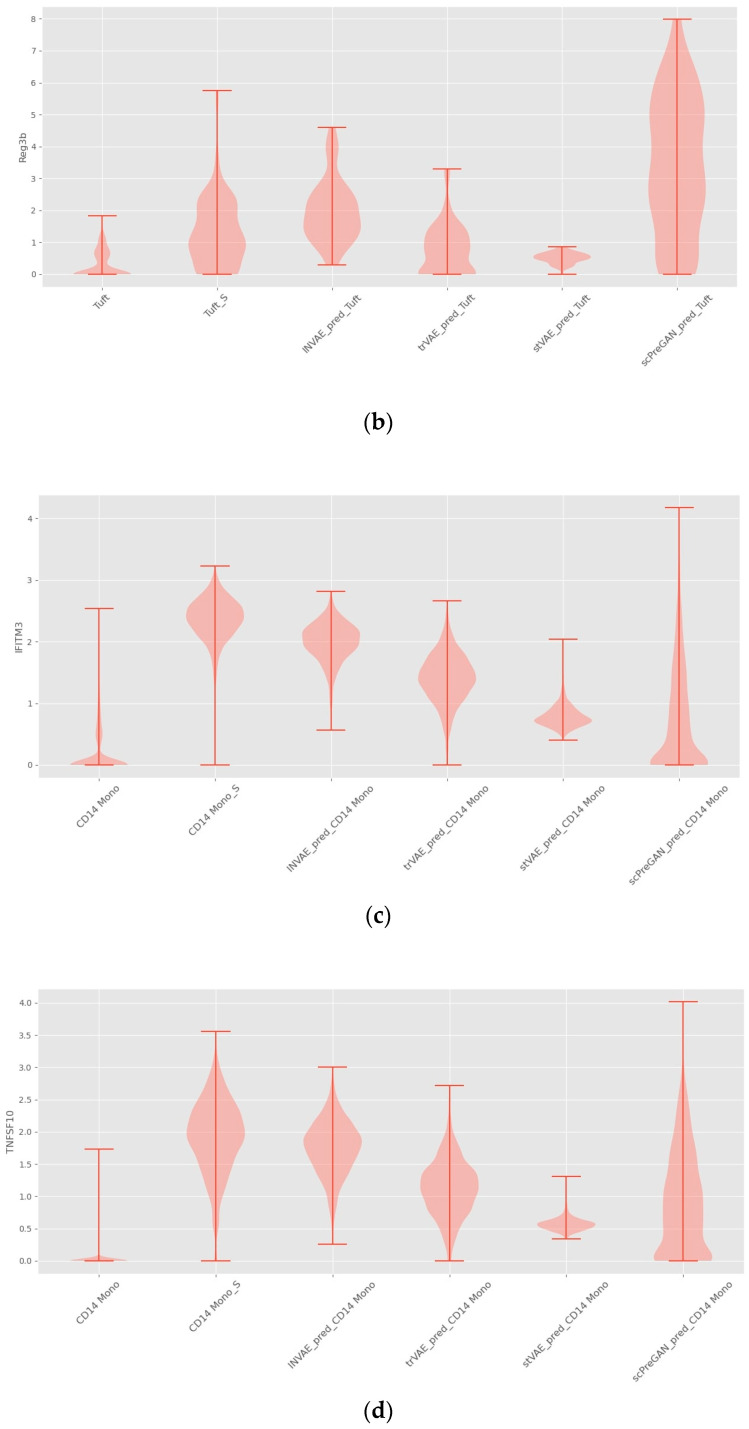
Violin plots with random sampling of the four model-predicted gene distributions and the true predicted gene distributions, cell names with the suffix “S” indicate perturbation conditions. (**a**) Violin plot of *Avil* in the Haber dataset. (**b**) Violin plot of *Reg3b* in the Haber dataset. (**c**) Violin plot of *IFITM3* from the Kang dataset. (**d**) Violin plot of *TNFSF10* from the Kang dataset.

Figure 7a shows that trVAE and scPreGAN are tricked into predicting nearly zero gene expression levels perturbed by Tuft Avil, possibly because the two models were influenced by the perturbed *Avil* expression levels of reference cells, which had nearly zero *Avil* expression levels (See Figure 6a). Specifically, as shown in Figure 7b, INVAE and trVAE captured similar responses of Tuft and Endocrine in *Reg3b* regulation after H.poly infection, whereas the other two comparative models failed to capture this relationship. In Figure 7c,d, we can find that the three baseline models are more likely to give the prediction results of the average value of the gene expression level of the reference cells (see Figure 6c,d), while INVAE can give more accurate prediction results.

### 4.4. Ablation Study

To predict the state of the cell after perturbation, we must map the cell from one condition to another, for which INVAE constructs a homogeneous space and finds the mapping relationship between different condition spaces. In order to avoid compromising the correctness of the mapping relationship learned by the model in the process of learning target cell information, a training method inspired by transfer learning is adopted. We conduct an ablation study to evaluate the contribution of uncorrelated filtering. As above, we remove decoder 1 and treat all latent variables as condition-specific features of variables. We calculated the average R-squared value of all genes and the top 100 DEGs of the three datasets, and the results are shown in Table 5.

As shown in Table 5, irrelevance filtering greatly improves the prediction accuracy and removes information that is useless for prediction, so that the model only focuses on the features that change after perturbation. Irrelevance filtering also reduces information differences between cell types, as shown in Figure 5b,d, which makes it easier to model homogeneous spaces. Training models using methods inspired by transfer learning can lead to better and more stable predictions.

### 4.5. Model Convergence Time

**Figure 8:**
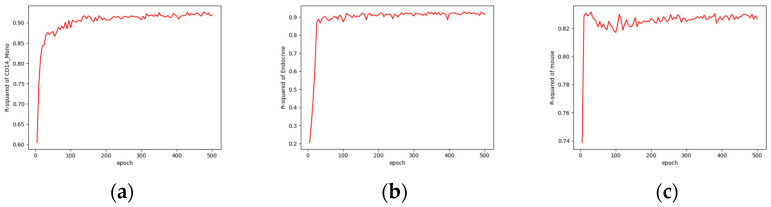
Comparisons of model convergence during training using Colab: (**a**) convergence of the CD14 mono prediction model; (**b**) convergence of the endocrine prediction model; (**c**) convergence of the mouse prediction model.

Figure 8 illustrates the convergence of the model during training for one of the three cell types from the three datasets. These three models were trained using the free resources (T4) available on Colab, utilizing our open-source code at https://github.com/LiouCharlie/INVAE/tree/main. We configured the models to train for 500 epochs, with each epoch taking only 2 to 3 s. As shown in Figure 8, our proposed method enables rapid convergence of the model to stable predictive outcomes.

## 5. Conclusions

Due to the huge difference in biological information between different cell types, the domains of reference cells and target cells violate the independent and identically distributed (i.i.d.) assumption of classical machine learning, resulting in a sharp drop in the accuracy of generating perturbed targets for one-to-one control of target cells [14]. In computer vision, out-of-distribution (OOD) problems are often addressed by finding domain invariance [26], aiming to find invariant information shared by different domains. However, reference data can be divided into multiple subdomains according to different cell types, and different subdomains may carry different information useful for predicting cellular responses, and using only the same features in each cell type may not yield the most accurate result prediction.

This paper proposes a new approach to this problem. By appropriately navigating different types of information into different variables, we can construct a homogeneous feature space, embed the prediction unit into the control condition at the appropriate position in the homogeneous feature space, and continue to map to the corresponding position in the perturbation condition space, so that the model can gather all the useful information of each subdomain to make accurate predictions, instead of just capturing information shared by all sub-domains or using information from only one sub-domain information. We further show that filtering out unfavorable information not only improves the prediction accuracy but also makes the prediction results more interpretable. We hope that this work will increase the productivity of biological researchers through its high predictive power of cellular responses and its ability to show possible similarities in predicting how cells respond to different cell types of drugs or infections.

## Figures and Tables

**Table 1:** Detailed parameters of INVAE encoder.

Name	Operation	Kernel Dim.	Dropout	Activation	Input
Input	-	input_dim	-	-	-
Conditions	-	1	-	-	-
Cell types	-	1	-	-	-
FC-1	FC	800	√	ReLU	[input, conditions, cell types]
FC-2	FC	800	√	ReLU	FC-1
FC-3	FC	128	√	ReLU	FC-2
Output s&c	FC	30 + 30	-	-	FC-3

**Table 2:** Detailed parameters of INVAE decoder 1.

Name	Operation	Kernel Dim.	Dropout	Activation	Input
FC-1	FC	128	√	ReLU	[Encoder output c, Cell types]
FC-2	FC	800	√	ReLU	FC-1
FC-3	FC	800	√	ReLU	FC-2
Output	FC	input_dim	-	-	FC-3

**Table 3:** Detailed parameters of INVAE projection layer.

Name	Operation	Kernel Dim.	Dropout	Activation	Input
Output	FC	128	√	ReLU	[Encoder output s, conditions]

**Table 4:** Detailed parameters of INVAE decoder 2.

Name	Operation	Kernel Dim.	Dropout	Activation	Input
FC-1	FC	800	√	ReLU	Projection layer output
FC-2	FC	800	√	ReLU	FC-1
Output	FC	input_dim	-	-	FC-2
Optimizer	Adam				
Learning rate	0.001				
Dropout rate	0.2				

**Table 5:** Results of an ablation study.

	INVAE	w/o Training Method	w/o Noise Filer
All	0.920	0.902	0.770
DEGs 100	0.786	0.734	0.588

## Data Availability

All datasets used in this study are publicly available.
